# Non-Adaptive MR-Guided Radiotherapy for Prostate SBRT: Less Time, Equal Results

**DOI:** 10.3390/jcm10153396

**Published:** 2021-07-30

**Authors:** Maria L. Sandoval, Irini Youssef, Kujtim Latifi, G. Daniel Grass, Javier Torres-Roca, Stephen Rosenberg, Kosj Yamoah, Peter A. Johnstone

**Affiliations:** 1 Department of Radiation Oncology, H. Lee Moffitt Cancer Center and Research Institute, Tampa, FL 33612, USA; Maria.sandoval@moffitt.org (M.L.S.); Kujtim.latifi@moffitt.org (K.L.); Daniel.grass@moffitt.org (G.D.G.); javier.torresroca@moffitt.org (J.T.-R.); Stephen.rosenberg@moffitt.org (S.R.); kosj.yamoah@moffitt.org (K.Y.); 2 Department of Radiation Oncology, SUNY Downstate Health Sciences University, Brooklyn, NY 11203, USA; irini.youssef@downstate.edu

**Keywords:** prostate, MR-guided, SBRT, non-adaptive

## Abstract

Background: The use of stereotactic body radiation therapy (SBRT) is widely utilized for treatment of localized prostate cancer. Magnetic-resonance-guided radiotherapy (MRgRT) was introduced in 2014 and has recently been implemented in SBRT for prostate cancer as it provides an opportunity for smaller margins and adaptive daily planning. Currently, the only publications of MRgRT for prostate SBRT describe European clinical experiences which utilized adaptive planning. However, adaptive planning adds significantly to the time required for daily treatment. Objectives: Since prostate SBRT has demonstrated acceptable toxicity for several years, we did not consider daily adaptation critical to the process of prostate SBRT. After Institutional Review Board approval, we analyzed and now report our experience using MRgRT without adaptation. Methods: Between 25 September 2019 and 21 December 2020, 35 consecutive patients were treated with MRgRT prostate SBRT at our center. Patients treated with MRgRT included favorable intermediate risk (43%) and unfavorable intermediate risk (54%), and only one patient had low-risk prostate cancer. Nine patients (25%) received adjuvant leuprolide for a median of 4.5 months (range 4–6 m). Our clinical pathway allows for a maximum prostate gland volume of 60 cc; median prostate volume of this cohort was 35.0 cc (range 17–58.4 cc). Median pre-treatment PSA was 6.30 (range 2.55–16.77). Each patient was treated with 36.25 Gy delivered in five fractions over 2 weeks with urethral sparing to a maximal dose of 35 Gy. Target volumes included the prostate gland and proximal seminal vesicles with a 3 mm margin. Results: Median follow-up as of 26 May 2021 was 11.97 months (range 4.37–19.80). First follow-up data are available for all patients, with a median of 1.10 month from completion of treatment (0.63–3.40). The median PSA at first visit was 2.75 (range 0.02–9.00) with a median AUA symptom score of 9 (range 1–24). Second follow-up data are available for 34 patients at a median of 4.45 months (range 2.57–8.90). At second follow-up, the median PSA was 1.60 (range 0.02–5.40) with a median AUA symptom score of 6 (range 1–33). Seventeen patients had third follow-up data with a median of 9.77 months (range 4.70–12.33) after SBRT. The median PSA was 1.13 (range 0.02–4.73) with an AUA score of 9 (2–22) at the third follow-up. We observed a statistically significant decrease in PSA between pre-treatment and at first follow-up (*p* < 0.005). The most common toxicity was grade 2 urethritis, managed in all cases by tamsulosin. One patient developed grade 2 tenesmus relieved by topical steroids. No cases of grade ≥ 3 toxicity were seen in our patient population. Conclusions: By avoiding the extra time required for plan adaptation, MRgRT without daily adaptation allows for successful prostate SBRT with manageable toxicity. We continue to reserve our limited adaptive treatment slots for preoperative pancreatic and ultra-central lung SBRT patients, which require time-intensive respiratory gating and adaptive planning.

## 1. Introduction

Treatment options for localized prostate cancer include surgery or radiotherapy and even active surveillance for patients with low-risk and favorable intermediate-risk disease [1]. As radiotherapy techniques have improved from three-dimensional conformal to intensity-modulated and image-guided (IMRT-IGRT), there is a need to limit toxicity while delivering higher radiation doses. There has been a shift toward hypofractionation (HF) and ultrahypofractionation (UHF) approaches that take advantage of the low α/β ratio for prostate cancer. These strategies have changed treatment paradigms in both Europe [2,3] and the United States [4].

The use of UHF or stereotactic body radiation therapy (SBRT) is widely utilized for treatment of localized prostate cancer and has increased significantly in the United States [5]. Since its implementation, the early and late gastrointestinal (GI) and genitourinary (GU) toxicities have become points of emphasis and have been the focus of several studies [6,7,8]. Magnetic-resonance-guided radiotherapy (MRgRT) was introduced in 2014 and has recently been implemented in SBRT for prostate cancer, with the goal of increasing visualization of soft tissues, leading to potentially smaller margins and adaptive daily planning which could minimize GI and GU toxicity and allow for dose escalation. MR also allows improved visualization within the prostate for urethral sparing and simultaneous integrated boost to gross disease.

Currently, there are two publications of MRgRT for prostate SBRT that have dealt with European clinical experiences [9,10], and all the reported cases had undergone adaptive planning with daily treatment. Results from both studies suggest that this approach was safe and well tolerated by patients. One of the rationales for daily adaptive treatment is that this allows tracking changes in prostate position and motion as well as changes in location of adjacent OARs which may contribute to toxicity seen in other reports [11]. However, adaptive planning adds significantly to the time required for daily treatment with a median time per treatment of 1 h [9], and prostate SBRT has been done with manageable toxicity for several years. Given the opportunity for gating with MRgRT and improved soft-tissue visualization, our prostate SBRT program did not perform daily adaptation for treatments. We hypothesized that the opportunities afforded through tumor tracking and improved soft-tissue visualization, without daily adaptation, could lead to excellent tumor control with minimal toxicity.

## 2. Materials and Methods

### 2.1. Patient Eligibility

After obtaining IRB approval (MCC#20383), patients were identified from a database at our institution who were treated with MRgRT. Between 25 September 2019 and 21 December 2020, 35 patients were treated with MRgRT prostate SBRT at our institution and retrospectively analyzed. Patients considered candidates for prostate SBRT included low-risk disease and favorable and unfavorable intermediate-risk disease who had completed staging work-up including pelvic MRI or computed tomography (CT) and had no evidence of lymph node involvement or distant metastases. Use of androgen deprivation therapy (ADT) was permitted by discretion of the treating physician. Our clinical pathway allows for maximum prostate gland volume of 60 cc for SBRT.

### 2.2. MRgRT Planning and Treatment

Patients were instructed to have full bladder and empty rectum before simulation and treatments. Patients underwent MRI simulation in the 0.35T MRIdian System (ViewRay Inc., Mountain View, CA, USA) along with CT for dose calculation, both scans done in the supine position typically with MR first followed by CT. During MRI simulation, a balanced steady-state free progression (TrueFISP) imaging sequence was used to create images weighted by T2/T1 ratio. No additional immobilization devices were utilized during simulation. A representative slice of the lesion was contoured as a tracking structure on a real-time single-plane sagittal cine-MRI sequence at 4–8 frames per second, and a 3 mm gating structure was created, as previously described by our group [12].

Clinical target volume (CTV) included the prostate gland and proximal seminal vesicles with a 3 mm isotropic expansion. Organs at risk (OARs) included urethra, bladder, and rectum which were all contoured. Patients were treated to a total of 36.25 Gy in a total of 5 fractions of 7.25 Gy/fraction delivered every other day over a two-week period with simultaneous urethral sparing to limit the maximal dose to 35 Gy.

### 2.3. Study Endpoints

The primary endpoint of the current study was tumor control as determined by PSA monitoring. The secondary endpoint was patient-reported American Urological Association (AUA) symptom score questionnaire, and toxicity grade was evaluated using Common Terminology Criteria for Adverse Events (CTCAE) version 5.0.

### 2.4. Statistical Analysis

Summary statistics were used to describe prostate gland size, tumor stage (clinical T stage, clinical N stage, Gleason score), the use of ADT (yes, no), and PSA (prior to and on follow-up). We used repeated-measures analysis of variance to test for changes in total PSA before radiation and after completion of therapy on two different follow-ups. A *p*-value of ≤0.05 was considered statistically significant. A post hoc pairwise comparison using the Bonferroni correction was used to explore differences between the different time periods.

## 3. Results

Between September 2019 and December 2020, a total of 35 patients were treated with MRgRT prostate SBRT. Median follow-up as of 26 May 2021 was 11.97 months (range 4.37–19.80). Patient characteristics are depicted in Table 1. The median age was 70 years old (range 51–82). Intermediate-risk prostate cancer was the predominant risk group in our cohort: 42.86% had favorable intermediate risk and 54.29% had unfavorable intermediate risk. Only one patient in our study had low-risk prostate cancer. The majority of the patients (94%) had T1c disease and only 2 out of 35 had T2 disease. Twenty-five percent of patients received concurrent short-term ADT: five patients received 4 months (14%), and four patients received 6 months (11%). Median prostate size for our cohort was 35 cc (range 17.0–58.4 cc).

The current study reports preliminary findings with a median of 1-year follow-up from completion of RT, and therefore not all patients have all follow-up data points. All patients in our cohort have first follow-up data, with a median time from completion of RT of 1.10 months (range 0.63–3.40). Second follow-up data are available for 34 patients with a median follow-up time of 4.45 months (2.57–8.90), and third follow-up is available on 17 patients with a median of 9.77 months (4.70–12.33) from completion of RT (Table 1).

**Figure 1:**
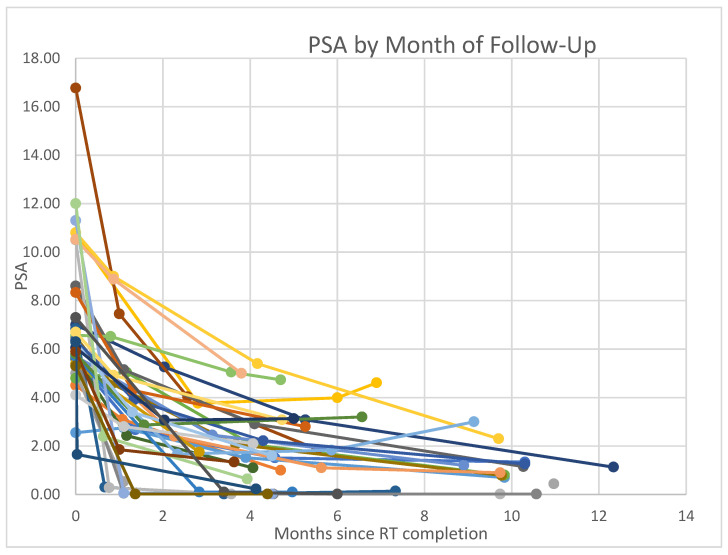
PSA trends across time.

In terms of PSA control, the median pre-treatment PSA was 6.30 ng/mL (range 2.55–16.77). As indicated in Figure 1, the PSA levels decreased by months from RT completion. Median decrease from pre-treatment PSA to PSA at first follow-up is 3.45 ng/mL (range 0.24–11.24 ng/mL). On 33 patients with second follow-up PSA available, median PSA decrease was 4.66 ng/mL (range 1.03–12.74 ng/mL) from pre-treatment levels. Seventeen patients had PSA levels available at third follow-up, with a median PSA decrease of 5.68 ng/mL (range 1.8–14.9 ng/mL) from pre-treatment levels. A repeated-measures ANOVA determined that decrease in PSA across the different time points was statistically significant (*p* < 0.0005). A post hoc pairwise comparison using the Bonferroni correction showed a decrease in PSA between pre-treatment PSA and PSA at first follow-up, as well as PSA at first follow-up and follow-ups 2 and 3 (*p* < 0.005). However, there was no statistically significant difference between PSA at second and third follow-up (*p* = 0.1).

**Figure 2:**
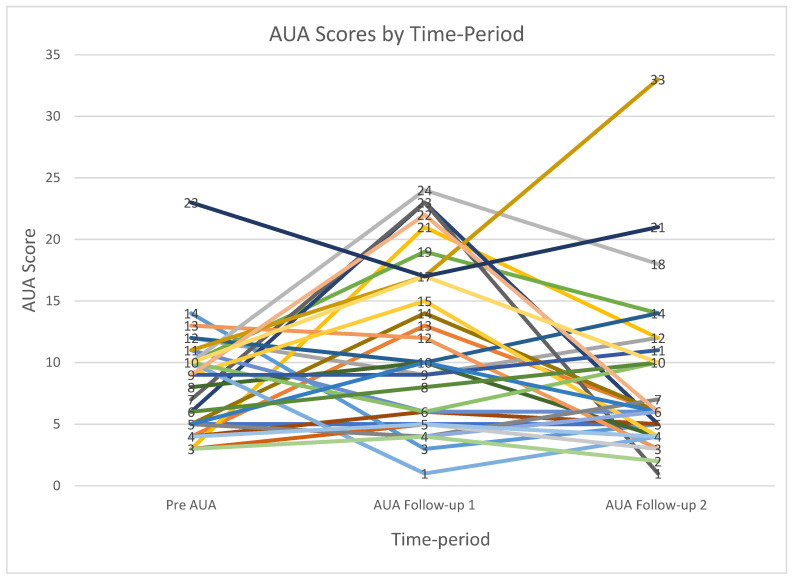
AUA trends across follow-up visits.

Toxicity was assessed using patient-reported AUA scores, and the median pre-treatment AUA was 7 (range 1–23). There were no grade 3 or higher toxicities in our patient population. Grade 1–2 urethritis was primarily seen during first follow-up appointment in 14 out 35 (40%) of patients, whereas it was only seen in 6 out of 34 (18%) patients on second follow-up and in 1 out of 17 (5.8%) on third follow-up. For analysis of trends, we used AUA scores for pre-treatment and follow-ups 1 and 2 from 27 patients that had complete data scores for these time points. Within this cohort of patients, there was an expected increase in the AUA score at first follow-up with a mean of 14.58, and it gradually decreased back to baseline in subsequent follow-ups (mean 7.75 at second follow-up) (Figure 2). A repeated-measures ANOVA determined that mean AUA values differed significantly across the three time points (*p* = 0.04).

## 4. Discussion

MRgRT with or without daily adaptation is a novel but rapidly expanding technology that has several aspects that make it particularly beneficial for treatment of prostate cancer. First, visualization of soft tissues such as the prostate is better in MRI than CT, and this can translate to smaller, more precise CTVs [13,14]. Additionally, due to better visualization and tracking, fiducials are not required hence sparing the patient from an invasive procedure [13]. The use of MRgRT has also been demonstrated in a metastatic setting and demonstrated good feasibility [15].

This study adds to the growing body of evidence supporting the use of MRgRT in the treatment of localized prostate cancer as an effective and safe strategy in selected patients. For patients with low risk and favorable and unfavorable intermediate risk with disease localized to prostate and no radiographic evidence of nodal involvement, non-adaptive MRgRT led to a significant decrease in PSA as early as 1 month after treatment (*p* < 0.005) (Figure 1). Additionally, our preliminary data show that this response is maintained over the three follow-up periods through almost 10 months.

With regards to toxicity, our results are in agreement with previously published literature. Bruynzeel et al. reported a maximum cumulative grade ≥ 2 early GU and GI toxicity of 23.8% and 5.0% using CTCAE and RTOG criteria, toxicity peaking shortly after the last fraction and resolving within the first 6 weeks of follow-up [10]. Similarly, Alongi et al. reported minimal toxicity with no grade ≥ 3 events [9]. Both studies reported radiation cystitis and proctitis as the most common toxicities observed in their patient populations [9,10]. Recent data suggest that a rectal spacer may further reduce symptoms using the physical functioning item of the EORT QLQ-C30 questionnaire [16]; we will await further clinical trial data.

We observed that grade 1–2 urethritis was primarily seen during first follow-up in 40% of patients and subsequently decreased on long-term follow-up, being present in 18% of patients on second follow-up and 6% at third follow-up. Since this study reports preliminary data, we do not have follow-up data for all patients, and therefore we did a sub-analysis of patients who had complete data across all three follow-up time points. The trend that we observed in this cohort demonstrates that there is a sharp but expected increase in the AUA score from pre-treatment to first follow-up, from 7.41 at baseline to 14.58. However, on second follow-up, we saw a significant decrease, with an AUA score of 7.75 (*p* = 0.043) (Figure 2). Of note, in our patient cohort, we did not see any grade ≥3 toxicities. The most common toxicities were grade 1–2 urethritis, managed by tamsulosin, and a single case of grade 2 tenesmus, managed by steroid suppositories.

With the advancement of different radiation techniques, the role of MRgRT for prostate cancer remains to be established. However, it offers exciting avenues for improvement in oncologic outcomes and minimization of acute and long-term toxicity. Adaptive MRgRT is an extremely involved process that requires a high number of person-hours since it involves the attention of a radiation oncologist, multiple radiographers, and a physicist thus potentially limiting its use [14]. In a recent prospective study of adaptive MRgRT, the reported fraction time ranged between 34 and 86 min [9], which highlights the time-intensive nature of this approach. At our institution, we have a single MRI-linac, and optimization of resources is essential. Given the breadth of literature on safety and efficacy of prostate SBRT, we reported our experience using non-adaptive MRgRT and by so doing, reserved the longer adaptive slots for treatment of other malignancies such as ultra-central lung and pancreatic cancer [11]. Limitations of our study include a small sample size, short follow-up times, and a single-institution patient cohort.

## 5. Conclusions

To the best of our knowledge, this is the first report describing non-adaptive MRgRT for prostate SBRT. By avoiding the extra time required for plan adaptation, MRgRT allows for successful prostate SBRT with good PSA response and manageable toxicity as demonstrated by patient-reported AUA scores. The current study demonstrates that this treatment approach is effective and allows us to reserve the fewer adaptive treatment slots for appropriate adaptive cases.

## Figures and Tables

**Table 1:** Baseline patient characteristics.

Stage	No.	%
T1c	33	94.29
T2	2	5.71
**Gleason Grade Group**	**No.**	**%**
1	1	2.86
2	22	62.86
3	12	34.29
**ADT**	9	25.71
**Duration**	**No.**	
4 months	5	
6 months	4	
**Prostate Gland Size**		
Median	35.0	
Range	17.0–58.4	
**Median Follow Up (mos)**	11.97	
1st Follow Up (mos)	1.10	0.63–3.40
2nd Follow Up (mos)	4.45	2.57–8.90
3rd Follow Up (mos)	9.77	4.70–12.33

## Data Availability

Institutional database used for patients in this cohort is not publicly available.
